# Diagnosis of Typical Apple Diseases: A Deep Learning Method Based on Multi-Scale Dense Classification Network

**DOI:** 10.3389/fpls.2021.698474

**Published:** 2021-10-01

**Authors:** Yunong Tian, En Li, Zize Liang, Min Tan, Xiongkui He

**Affiliations:** ^1^State Key Laboratory of Management and Control for Complex Systems, Institute of Automation, Chinese Academy of Sciences, Beijing, China; ^2^School of Artificial Intelligence, University of Chinese Academy of Sciences, Beijing, China; ^3^College of Science, China Agricultural University, Beijing, China

**Keywords:** apple disease diagnosis, Cycle-GAN, Multi-scale connection, DenseNet, deep learning

## Abstract

Disease has always been one of the main reasons for the decline of apple quality and yield, which directly harms the development of agricultural economy. Therefore, precise diagnosis of apple diseases and correct decision making are important measures to reduce agricultural losses and promote economic growth. In this paper, a novel Multi-scale Dense classification network is adopted to realize the diagnosis of 11 types of images, including healthy and diseased apple fruits and leaves. The diagnosis of different kinds of diseases and the same disease with different grades was accomplished. First of all, to solve the problem of insufficient images of anthracnose and ring rot, Cycle-GAN algorithm was applied to achieve dataset expansion on the basis of traditional image augmentation methods. Cycle-GAN learned the image characteristics of healthy apples and diseased apples to generate anthracnose and ring rot lesions on the surface of healthy apple fruits. The diseased apple images generated by Cycle-GAN were added to the training set, which improved the diagnosis performance compared with other traditional image augmentation methods. Subsequently, DenseNet and Multi-scale connection were adopted to establish two kinds of models, Multi-scale Dense Inception-V4 and Multi-scale Dense Inception-Resnet-V2, which facilitated the reuse of image features of the bottom layers in the classification neural networks. Both models accomplished the diagnosis of 11 different types of images. The classification accuracy was 94.31 and 94.74%, respectively, which exceeded DenseNet-121 network and reached the state-of-the-art level.

## 1. Introduction and Related Works

Nowadays, apple is one of the most widely grown, highly productive and popular fruits in the world. The quality of fruits directly determines the economic development of the apple plantation industry. However, different kinds of diseases have always been one of the major causes for the decline in apple quality and yield, and directly harm the development of agricultural economy. Therefore, precise diagnosis of apple diseases and correct treatments are important measures to alleviate agricultural losses and promote economic development.

At present, the diagnosis of most plant diseases still depends on farmers. However, as the image features of some diseases are similar, and there is no obvious boundary between different grades of the same disease, the artificial diagnosis results might present a large deviation. This poses a challenge to disease management. Moreover, due to the random occurrence, some diseases cannot be found in time. This will affect the quality and yield of fruits, and then harm the development of agricultural economy. Therefore, it is increasingly significant to use computer vision and deep learning methods to achieve automatic and precise disease diagnosis. With the continuous maturity of agricultural Internet of Things (IoT) technology, sensor networks and information perception systems are increasingly employed in agricultural industry (Khanna and Kaur, 2019; Muangprathub et al., 2019). This plays a key role in obtaining timely disease information and making precise decisions on disease prevention. In terms of plant disease information processing, image information has become a common basis for identifying disease types and judging the grades of the disease (Sankaran et al., 2010; Zhang J. et al., 2019).

In traditional image processing, binarization is often utilized to extract lesion areas. These methods can process images with simple backgrounds, but they cannot be readily applied in complex image background due to the lack of available image features. In the past few years, traditional image processing techniques have been widely adopted in plant disease identification and detection. Zou et al. (2010) used a multi-threshold segmentation technique to detect the lesion area of apples. This method can extract the lesion area on the surface of the apple, but the disease type cannot be diagnosed. Zarifneshat et al. (2012) adopted artificial neural network (ANN) to predict the volume of the apple lesion. Rumpf et al. (2010) made use of the support vector machine (SVM) to classify the multi-spectral images of lesions and realized the diagnosis of plant diseases. Omrani et al. (2014) used SVM and ANN to diagnose diseases such as apple spot alternaria and black spot, and compared the performance of the two models. In our previous research, a feedback neural network optimized by a genetic algorithm was employed to segment apple fruit regions in complex environments (Tian et al., 2018). The SVM method was then used to extract the lesion areas in the fruit images. This method realized the detection of apple diseases in a complex environment. Although traditional methods have achieved the diagnosis and detection of plant diseases in certain circumstances, they are not effective in complex environments. Moreover, the image processing time is relatively long, which cannot meet the demand for timely and accurate plant disease diagnosis.

With the continuous upgrading of processor computing power, artificial intelligence and machine learning technologies are favored in the field of smart agriculture (Patricio and Rieder, 2018). In terms of image processing of plant diseases, Mohanty et al. (2016) used AlexNet (Krizhevsky et al., 2012) and GoogLeNet (Szegedy et al., 2015) to realize the recognition of 14 crops and 26 diseases, with the accuracy of 99.35%. The dataset images used in this research are crop images with monotonous backgrounds, rather than taken in field. Amara et al. (2017) adopted LeNet (Lecun et al., 1998) as the backbone to identify banana leaf diseases under complex backgrounds and illumination conditions. Sladojevic et al. (2016) made use of CaffeNet (Jia et al., 2014) to classify 13 leaf diseases, and the accuracy reached 96.3%. Jiang et al. (2019) proposed an INAR-SSD method for real-time diagnosis and detection of 5 common apple leaf diseases, achieving 78.80% mean average precision and 23.13 frames per second. Zhang S.et al. (2019) proposed a global pooling dilated convolution neural network to diagnose 6 types of common cucumber leaf diseases with the accuracy of 94.65%, which is better than commonly used depth convolutional neural networks. Zhong and Zhao (2020) adopted DenseNet-121 (Huang et al., 2017) backbone to diagnose 6 types of apple leaf diseases. Three methods of regression, multi-label classification and attention loss function were employed for comparison on the basis of DenseNet-121. The accuracy reached 93.51, 93.31, and 93.71% respectively, achieving the state-of-the-art level. However, due to the phenomenon of image feature loss, it is difficult to improve the diagnostic accuracy merely by increasing the depth of network layers, which brings a challenge to meet the increasing demand for diagnosis.

The outstanding performance of convolutional neural networks is based on sufficient training data. During the acquisition of apple disease images, the randomness of disease occurrence makes it difficult to collect a large amount of data. Traditional image augmentation methods, such as rotation (Tan et al., 2016), mirroring (Dyrmann et al., 2016), translation (Sladojevic et al., 2016), scale transformation (Bargoti and Underwood, 2016), brightness transformation, color transformation (Tian et al., 2019b) and image mosaic (Tian et al., 2020), have been applied in the process of dataset establishment. However, these methods do not change the super-pixel information of the image, and still retain the similarity of the color, brightness, texture, and other features between the adjacent pixels of the original image. In our previous research, the Cycle-GAN (Isola et al., 2018; Shen et al., 2019) method was employed to generate anthracnose lesion images on the surface of healthy apples (Tian et al., 2019a). This method effectively expanded the training set. However, in our previous work, only anthracnose images have been generated. Various kinds of diseases and different grades of the same disease need to be further processed to verify the feasibility of the algorithm.

In this paper, a Multi-scale Dense convolutional neural network backbone is designed for diagnosing 11 types of images including healthy and diseased apple fruits and leaves. The main contributions are as follows:

1. Cycle-GAN method was adopted to generate two diseases, anthracnose and ring rot, on the surface of healthy apples. This method completed the data augmentation in the super-pixel space.
2. A Multi-scale Dense neural network backbone for apple disease diagnosis was proposed. The effectiveness of existing classification models has been improved.
3. It was validated in the experiment that the proposed method outperformed other common disease diagnosis models, and achieved the state-of-the-art level.

The paper has been organized in the following manner. Section 2 introduces the materials and methods, including image acquisition, image augmentation, and apple disease diagnosis models. Section 3 concerns the experiments and the performance of the proposed methodologies. Finally, our conclusions are presented.

## 2. Materials and Methods

### 2.1. Dataset Preparation

#### 2.1.1. Composition of Image Dataset

**Figure 1:**
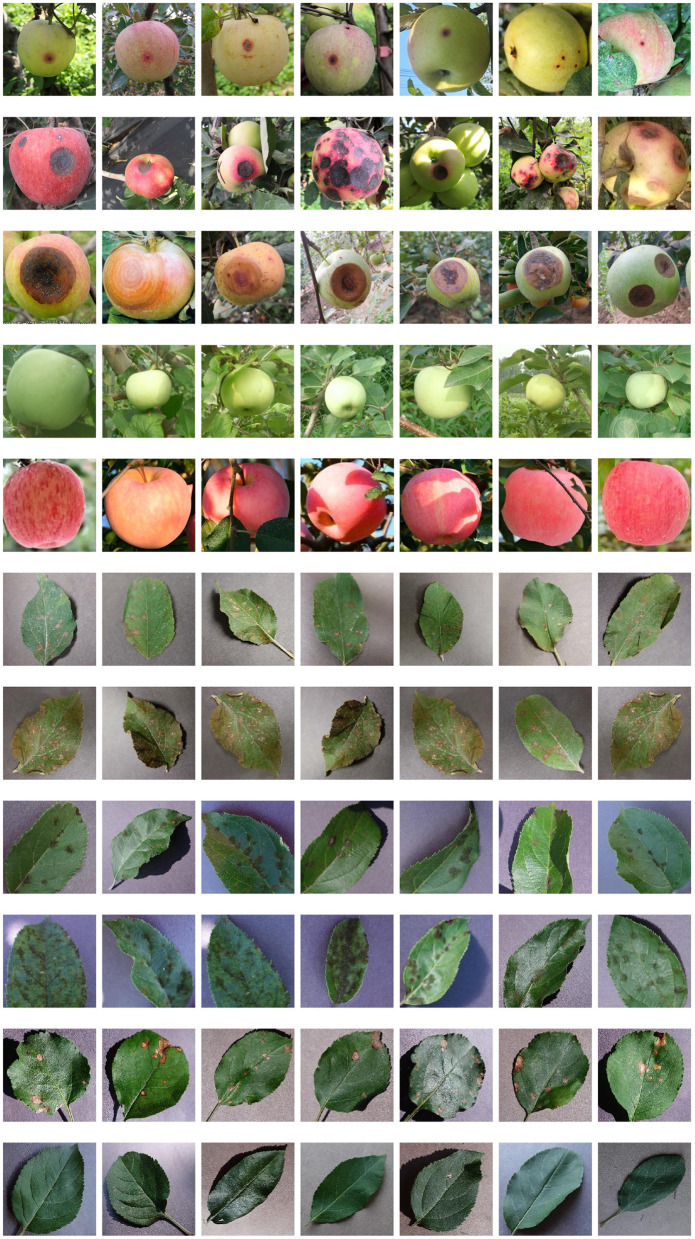
Some images of the dataset adopted in this paper, from **top** to **bottom**, are general anthracnose, serious anthracnose, ring rot, healthy green apple, healthy red apple, general cedar apple rust, serious cedar apple rust, general apple scab, serious apple scab, apple gray spot, healthy apple leaf.

The dataset employed in our research includes diseased leaf images, diseased fruit images, healthy leaf images, and healthy fruit images, as shown in Figure 1. The images of apple leaves come from Challenger-Plant-Disease-Recognition (https://gitee.com/cheng_xiao_yuan/AI-Challenger-Plant-Disease-Recognition). The leaf images are divided into six categories, including healthy apple leaf, general apple scab, serious apple scab, apple gray spot, general cedar apple rust, and serious cedar apple rust. The fruit images were collected in the field. These images include five categories, including healthy green apple fruit, healthy red apple fruit, general anthracnose, serious anthracnose, and ring rot. The number of original images collected is shown in Table 1. Due to the random occurrence of apple diseases, it is arduous to obtain abundant images of diseases in the field. Therefore, the number of collected apple images of anthracnose and ring rot is far less than that of other types of diseases, which cannot meet the training requirements of deep learning neural networks. Since some of the image features of anthracnose and ring rot are similar, and there is no clear boundary between different grades of the same disease, more images are needed to obtain rich image features. However, the images collected in the field were insufficient for training the deep neural networks.

**Table 1:** Categories, labels, and quantity of images in the dataset.

**Category**	**Label**	**Size of original datasets**	**Size of augmented datasets**
Healthy apple leaf	0	1,354	1,354
General apple scab	1	1,205	1,205
Serious apple scab	2	1,026	1,026
Apple gray spot	3	488	976
General cedar apple rust	4	972	972
Serious cedar apple rust	5	460	1,380
Healthy green apple fruit	6	1,360	1,360
Healthy red apple fruit	7	1,360	1,360
General anthracnose	8	78	1,014
Serious anthracnose	9	87	1,044
Ring rot	10	93	1,023

#### 2.1.2. Image Data Augmentation

**Figure 2:**
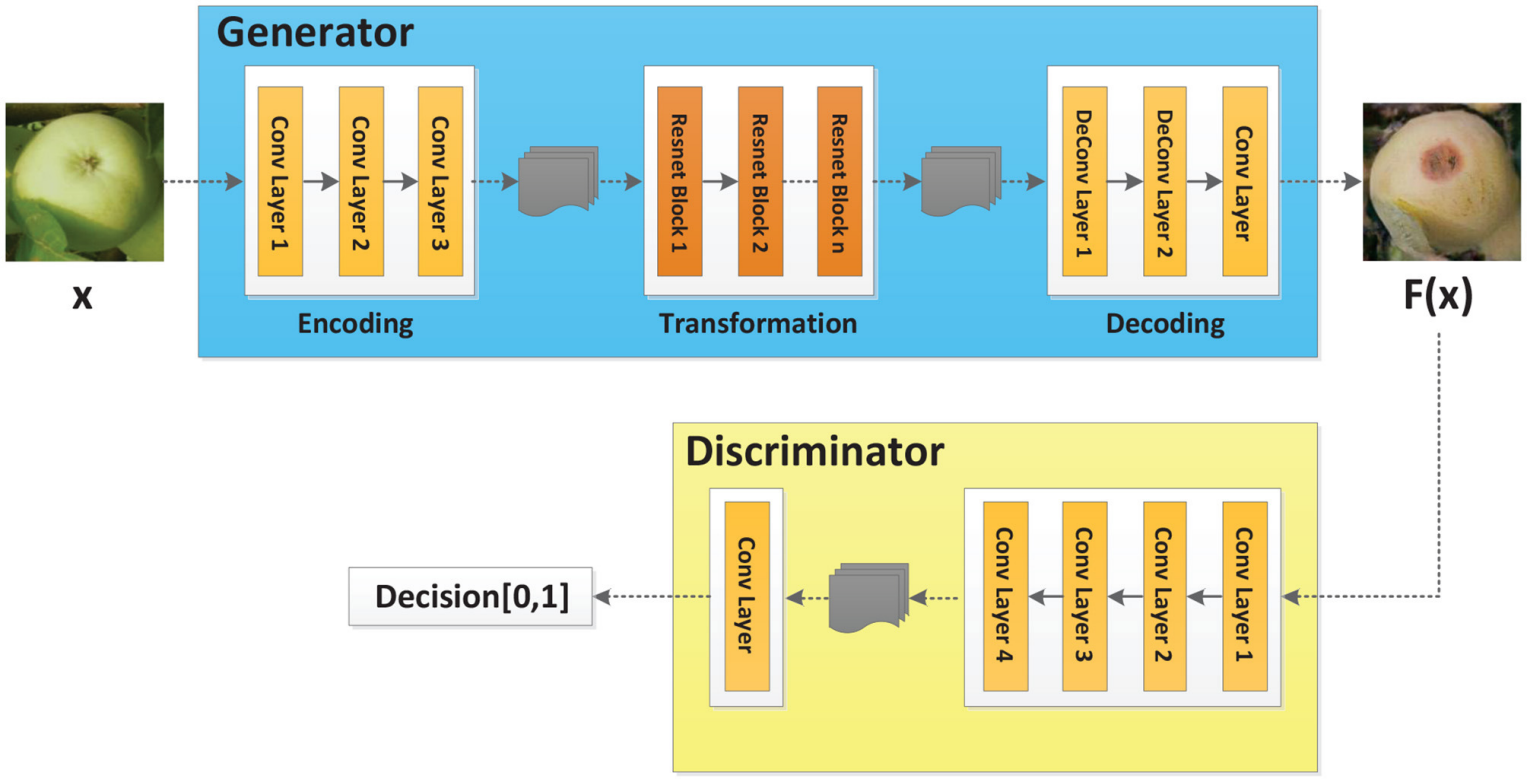
Cycle-GAN model.

In order to meet the demand for the number of images in the training set, Cycle-GAN (Isola et al., 2018) is adopted to further augment the image dataset upon traditional methods. Cycle-GAN algorithm can realize image style transfer by learning two different types of images. In other words, there are now two image sample spaces *X* and *Y*. *X* represents the image space of healthy apples, and *Y* represents the image space of diseased apples. The image in space *X* is expected to be converted to an image in space *Y*. The Cycle-GAN algorithm transforms the image *x* in space *X* into the image *F*(*x*) in space *Y* through the generator *F*. In order to determine whether the generated image *F*(*x*) is an image in space *Y*, the Cycle-GAN algorithm imports a discriminator *D*_*Y*_, which, combined with the generator *F*, constitutes an adversarial neural network, as shown in Figure 2. Finally, by learning the image features of healthy and diseased apples, the generator converts the healthy apple image into a diseased apple image. The discriminator determines that the generated image is in the diseased apple image dataset, which completes the image style conversion.

To remedy the insufficient number of gray spot and serious cedar apple rust images in apple leaf disease dataset, rotation and brightness transformation were adopted to augment the original image. According to the lack of diseased apple fruit images, brightness transformation, rotation transformation and mirror transformation were first exploited to generate abundant images. Subsequently, the Cycle-GAN technique was utilized to perform feature learning on general anthracnose, serious anthracnose, and ring rot images, then generate these three types of lesions on healthy apple images. After image style transformation, the generated images were provided to disease experts, who selected 500 images of each type of disease that could be employed as training samples. These generated images were added to the dataset. The dataset composition after image augmentation is shown in Table 1.

### 2.2. Multi-Scale Dense Classification Network

Deep learning network is an effective method to achieve target classification. In recent years, LeNet (Lecun et al., 1998), AlexNet (Krizhevsky et al., 2012), VGG (Simonyan and Zisserman, 2015), GoogLeNet (Szegedy et al., 2015), DenseNet (Huang et al., 2017), Inception (Szegedy et al., 2016), etc. have been widely used in target classification. Due to the complex backgrounds of apple disease images, the image features of different diseases are similar, and the distinction between different grades of the same disease is inconspicuous. Therefore, it is necessary to further improve the performance of the existing deep learning networks. In general, increasing the number of network layers is employed to improve the fitting ability of the network. However, with the deepening of the network layers, the image features of the bottom layers are gradually lost. This results in the inadequate usages of image features. Therefore, when a network reaches a certain depth, increasing the number of network layers cannot continue to improve its capability.

As the Inception series classification networks have shown superior performance on multiple classification tasks, the state-of-the-art neural networks Inception-V4 and Inception-ResNet-V2 (Szegedy et al., 2016) were adopted as the backbone in this paper. The idea of Multi-scale connection and DenseNet was introduced to construct new network models, achieving the purpose of promoting feature reuse and improving network performance.

#### 2.2.1. Inception-V4 and Inception-ResNet-V2

**Figure 3:**
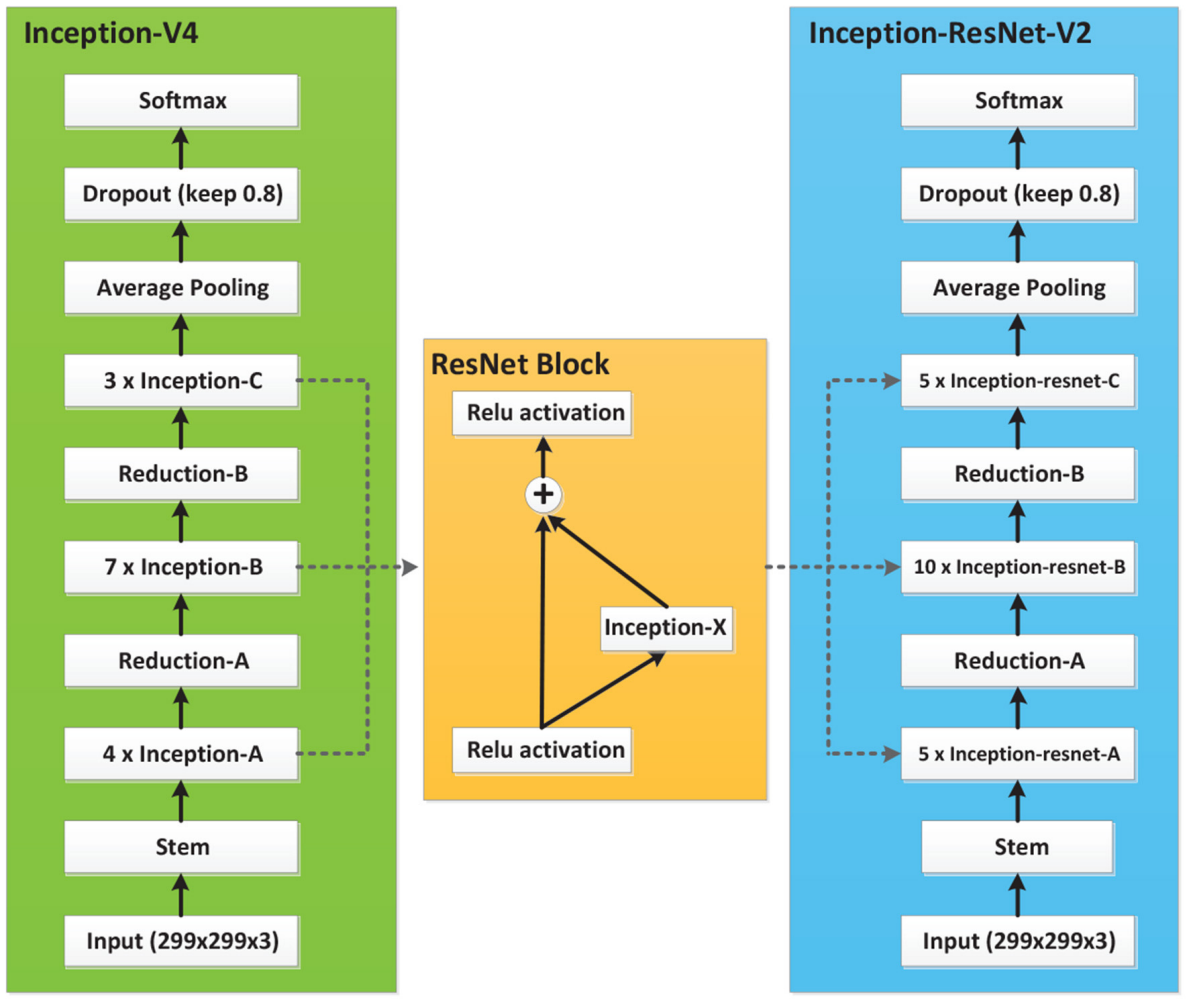
Network structures of Inception-V4 and Inception-ResNet-V2.

**Figure 4:**
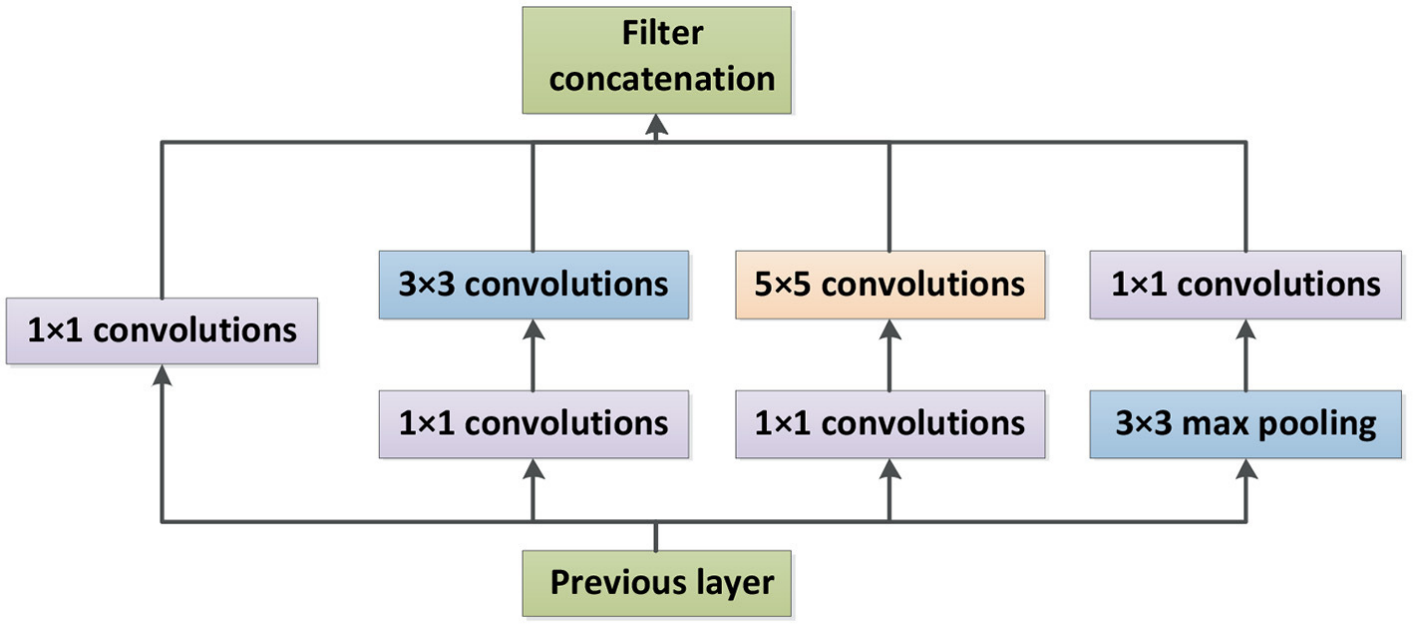
Inception module.

Inception-V4 and Inception-ResNet-V2 networks were optimized on the basis of Inception-V3 (Szegedy et al., 2016). The network structure of Inception is shown in Figure 3, where Stem is the basic structure of feature extraction, which consists of multiple convolution and pooling operations. The Inception-X module in the backbone learns image features through the feature transfer architecture of several parallel structures, which has a higher feature utilization rate than the previous Inception versions. One basic mechanism of the Inception module is shown as Figure 4. The entire Inception structure is strung together by several Inception modules. There are two main contributions of the Inception structure. One is to use 1 × 1 convolution to raise and lower the dimension.The other is to convolve and aggregate on multiple dimensions at the same time. Inception-ResNet-V2 introduces the ResNet module over inception-V4, which further improves the performance of the network.

#### 2.2.2. DenseNet

In order to make better use of the image features, DenseNet (Huang et al., 2017) structure was introduced into the networks. DenseNet realizes the concatenation of features in different depths by establishing dense connections between the front layers and the back layers, thereby achieving feature reuse.

**Figure 5:**
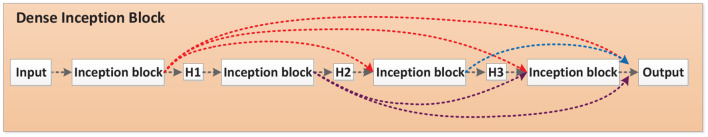
Dense Inception block.

Each Inception-(ResNet-)X module in the Inception-X layer and Inception-ResNet-X layer is called an Inception block. The four Inception-A modules in Inception-V4 are taken as an example, demonstrated in Figure 5. The output of the first Inception block is processed by the H1 function and then used as the input of the second Inception block. The output of the second Inception block is cascaded with the output of the previous block after H2 function operation. Their concatenation is the input of the third Inception block. The output of the third Inception block is cascaded with the output of the previous two Inception blocks after H3 function and then adopted as the input of the fourth Inception block. Finally, the outputs of all Inception blocks are spliced as the input of the subsequent network. In this paper, Hi stands for non-linear conversion function, which is a combination structure of batch normalization (BN), rectified linear units (RELU), and convolution (Conv), named BN-ReLU-Conv (1 × 1)-BN-ReLU-Conv (3 × 3).

#### 2.2.3. Multi-Scale Connection

**Figure 6:**
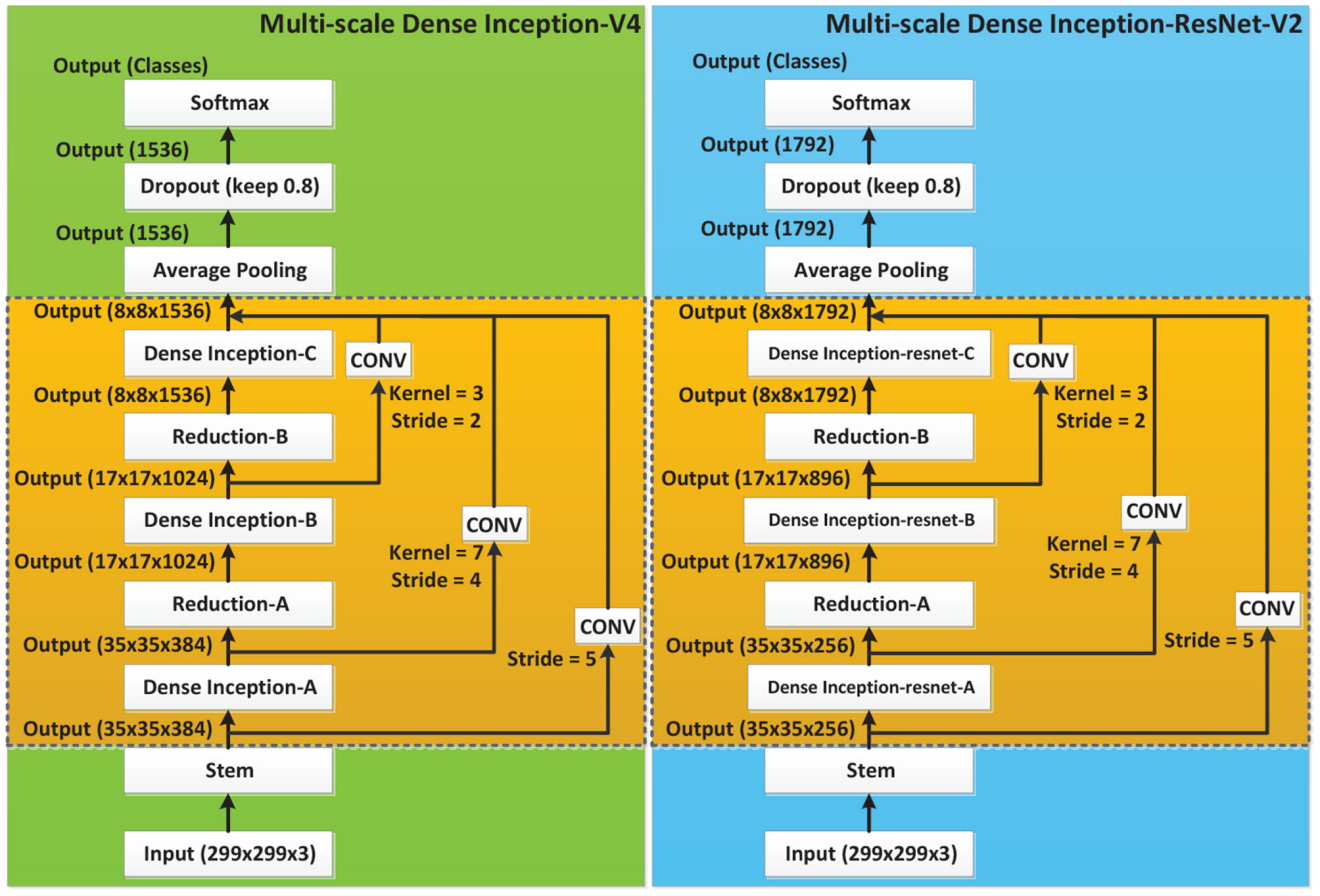
Multi-scale Dense neural networks.

On the basis of the Dense Inception block, a Multi-scale concatenation structure was designed to splice the different depths of the network, as shown in Figure 6. In the Multi-scale concatenation network model, the output of the Stem block is convolved and spliced with the output of each Dense Inception block. The concatenation result is sent to the average pooling layer as the input. Multi-scale connection enables network features of different depths to function in the final training, which further improves the utilization of bottom layer features.

#### 2.2.4. Details of Model Training

In this paper, the image dataset composition is shown in Table 1. In order to verify the performance of the deep learning models, 350 images of each disease were randomly selected from the augmented image dataset as the test set, and the remaining images as the training set. All diagnostic models were trained and tested on the IW4210-8G server. In the training process of Multi-scale Dense Inception-V4 and Multi-scale Dense Inception-ResNet-V2, the number of epoches was set to 4,000, the minimum batch was set to 16, the dropout was set to 0.8, the learning rate was set to 0.0001 and the decay rate was set to 0.95. The cross entropy function was used as the loss function as follows.


(1) $$\begin{array}{r} Loss=-\frac{1}{N}\overset{N}{\underset{i=1}{\sum }}\overset{M}{\underset{c=1}{\sum }}y_{ic}log\left(p_{ic}\right), \end{array}$$

where *N* is the number of training samples. *M* is the number of categories. *y*_*ic*_ is a symbolic function that is 1 when the true class of sample *i* is *c*, and 0 otherwise. *p*_*ic*_ is the prediction probability of sample *i* belonging to category *c*.

## 3. Experiments and Discussion

### 3.1. Evaluation of Cycle-GAN Method

**Figure 7:**
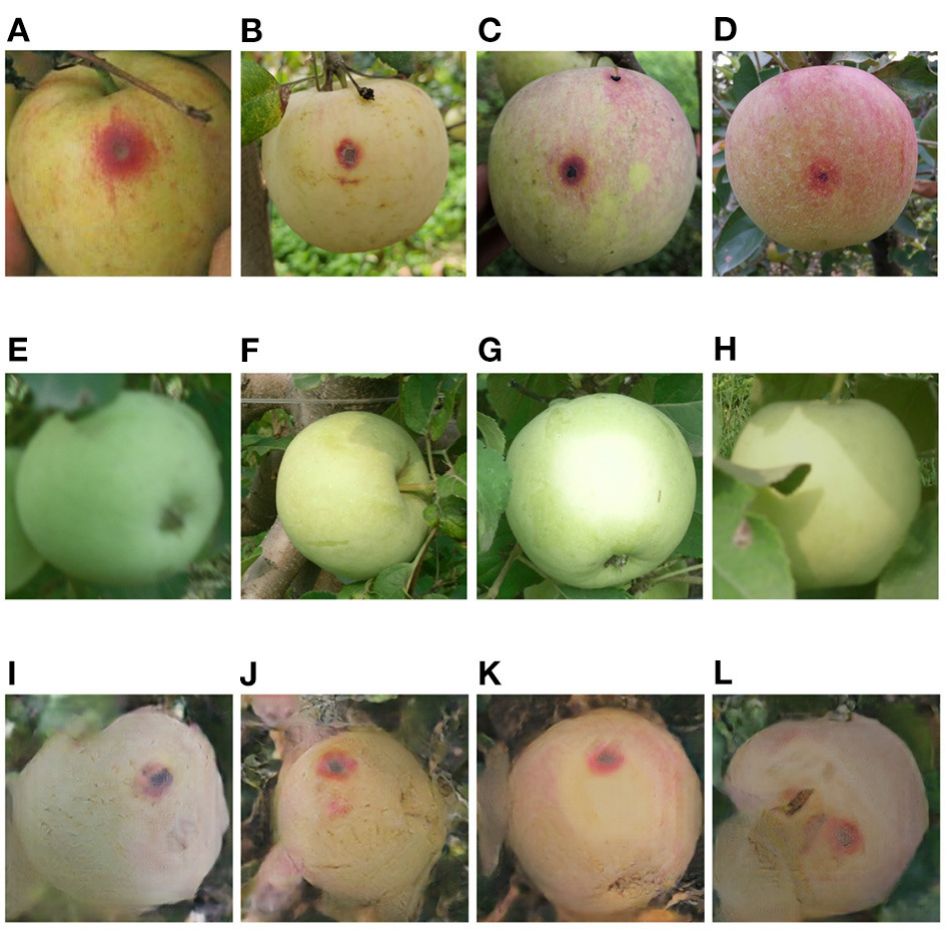
**(A–D)** General anthracnose apple images captured in field, **(E–H)** healthy apple images, **(I–L)** general anthracnose apple images converted from the above healthy apple images by the Cycle-GAN method.

**Figure 8:**
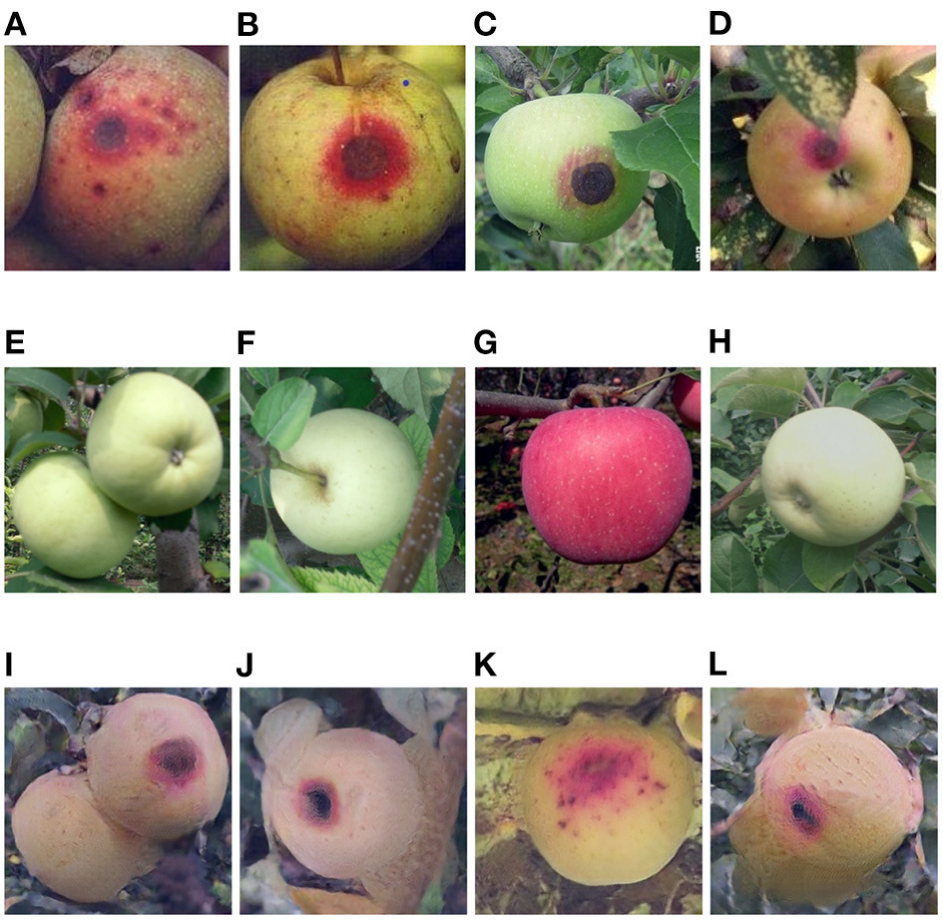
**(A–D)** Serious anthracnose apple images captured in field, **(E–H)** healthy apple images, **(I–L)** serious anthracnose apple images converted from the above healthy apple images by the Cycle-GAN method.

**Figure 9:**
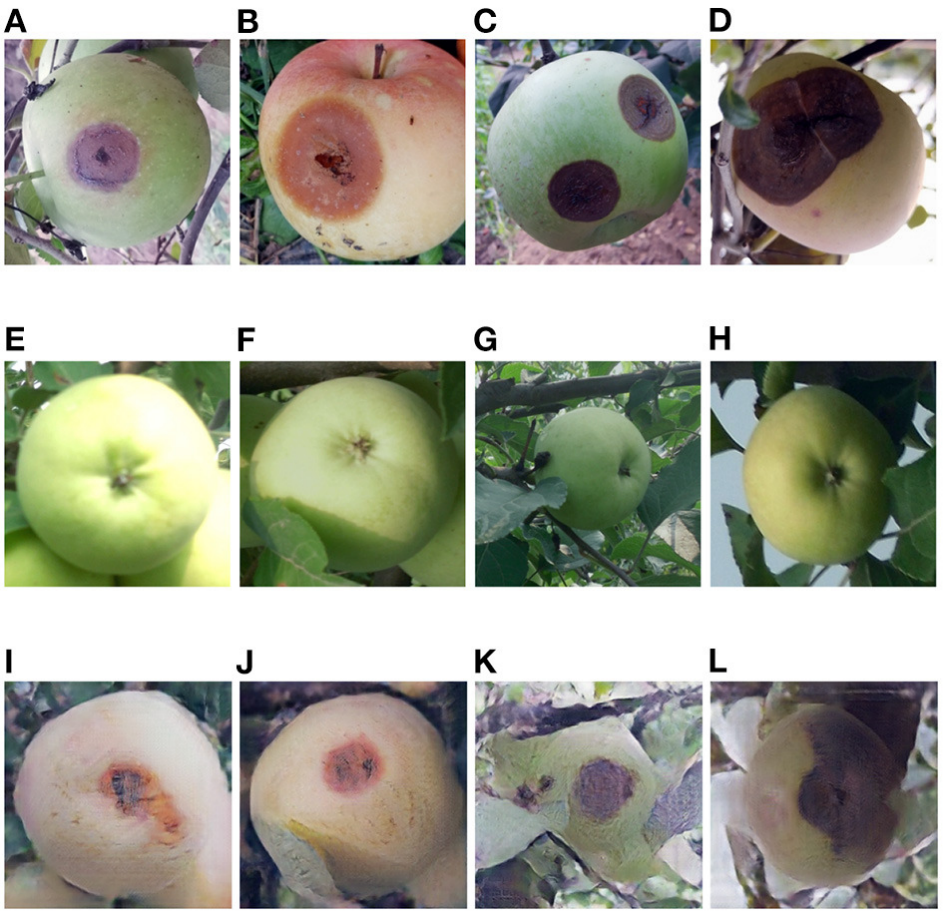
**(A–D)** Ring rot apple images captured in field, **(E–H)** healthy apple images, **(I–L)** ring rot apple images converted from the above healthy apple images by the Cycle-GAN method.

The Cycle-GAN method is introduced on the basis of traditional image augmentation methods to expand the image dataset. Some of the healthy and diseased apple images captured in the field and the diseased apple images generated by Cycle-GAN are shown in Figures 7–9. It can be seen that the generated diseased apple images are similar to the real images, and the disease characteristics are well-preserved. These images can be added to the dataset as the training samples. As described in section 2.1.2, these qualified images are selected from the generated images by experts. Images that can meet the requirements account for 73.1% of all generated images.

In order to better evaluate the Cycle-GAN method, two training sets were compared. One is a training set that contains images generated by Cycle-GAN. The other is the dataset that only adopts traditional augmentation techniques, such as brightness transformation, rotation transformation, and mirror transformation. The two training sets were employed to train the classification models, and the test results are shown in Table 2. According to the experimental results, Cycle-GAN method can generate new training samples in the super-pixel space and improve the richness of the training dataset more effectively than traditional image augmentation methods.

**Table 2:** Test results on different datasets.

**Model**	**Training set**	**Accuracy (%)**	**Precision (%)**	**Sensitivity (%)**	**F1 score (%)**
Multi-scale Dense Inception-V4	With Cycle-GAN	94.31	94.36	94.28	94.32
Multi-scale Dense Inception-ResNet-V2	With Cycle-GAN	94.74	94.71	95.03	94.86
Multi-scale Dense Inception-V4	Without Cycle-GAN	93.43	93.71	94.14	93.92
Multi-scale Dense Inception-ResNet-V2	Without Cycle-GAN	93.77	93.88	94.76	94.32

### 3.2. Comparison of Different Models

In order to evaluate the performance of the proposed Multi-scale Dense architecture, Inception-V4, Inception-ResNet-V2, Multi-scale Dense Inception-V4, Multi-scale Dense Inception-ResNet-V2, LeNet, AlexNet, VGG, and Densenet-121 were compared. All classification models were trained and tested on the IW4210-8G server. The training parameters of all models were unified. The experimental results of the eight models are shown in Table 3. It can be seen from Table 3 that there is no direct relationship between the training time and the size of the model, and the introduction of feature layer connection methods such as ResNet can accelerate the convergence of the neural network. The introduction of the Multi-scale connection and DenseNet has significantly improved the performance of Inception-V4 and Inception-ResNet-V2 models. The Multi-scale Dense Inception-ResNet-V2 model achieves the best accuracy, surpasses the Densenet-121 model, and realizes the state-of-the-art performance.

**Table 3:** Comparison of different models.

**Model**	**Accuracy (%)**	**Precision (%)**	**Sensitivity (%)**	**Size (M)**	**Training time (hours)**
Inception-V4	92.72	92.61	94.89	176	2.32
Inception-ResNet-V2	93.37	92.65	95.37	226	1.95
Multi-scale Dense Inception-V4	94.31	94.36	94.28	201	2.58
Multi-scale Dense Inception-ResNet-V2	94.74	94.71	95.03	255	2.11
LeNet	85.21	83.66	88.39	13	0.64
AlexNet	92.04	91.75	92.83	60	1.79
VGG	92.45	92.32	92.54	472	1.65
Densenet-121	93.68	93.66	95.12	46	3.29

### 3.3. Diagnosis of Different Diseases

In this paper, two models are proposed to classify images of 11 categories. The diagnosis results of different diseases are shown in Table 4. Accuracy1 is the diagnosis result of the Multi-scale Dense Inception-V4 model, and Accuracy2 is the diagnosis result of the Multi-scale Dense Inception-ResNet-V2 model. It can be seen from the results in Table 4 that healthy apple leaf and healthy apple fruits have the highest diagnostic accuracy. Because the distinction between general disease and serious diseases is not obvious, the accuracy of the diagnosis result is affected. In addition, the image features of apple gray spot and cedar apple rust have a certain similarity, so a few inaccurate diagnosis results are given among the test samples. However, combining the results of various categories, the two new models have shown superior results and can meet the diagnosis requirements.

**Table 4:** The diagnosis results of two models for different diseases.

**Disease**	**Accuracy1 (%)**	**Accuracy2 (%)**
Healthy apple leaf	95.63	95.82
General apple scab	93.18	93.21
Serious apple scab	93.45	93.79
Apple gray spot	92.11	92.27
General cedar apple rust	92.80	92.65
Serious cedar apple rust	93.12	93.86
Healthy green apple fruit	96.34	96.73
Healthy red apple fruit	96.22	96.75
General anthracnose	94.27	94.77
Serious anthracnose	94.15	94.45
Ring rot	93.97	94.38
Overall	94.31	94.74

### 3.4. Practical Application Scenarios

**Figure 10:**
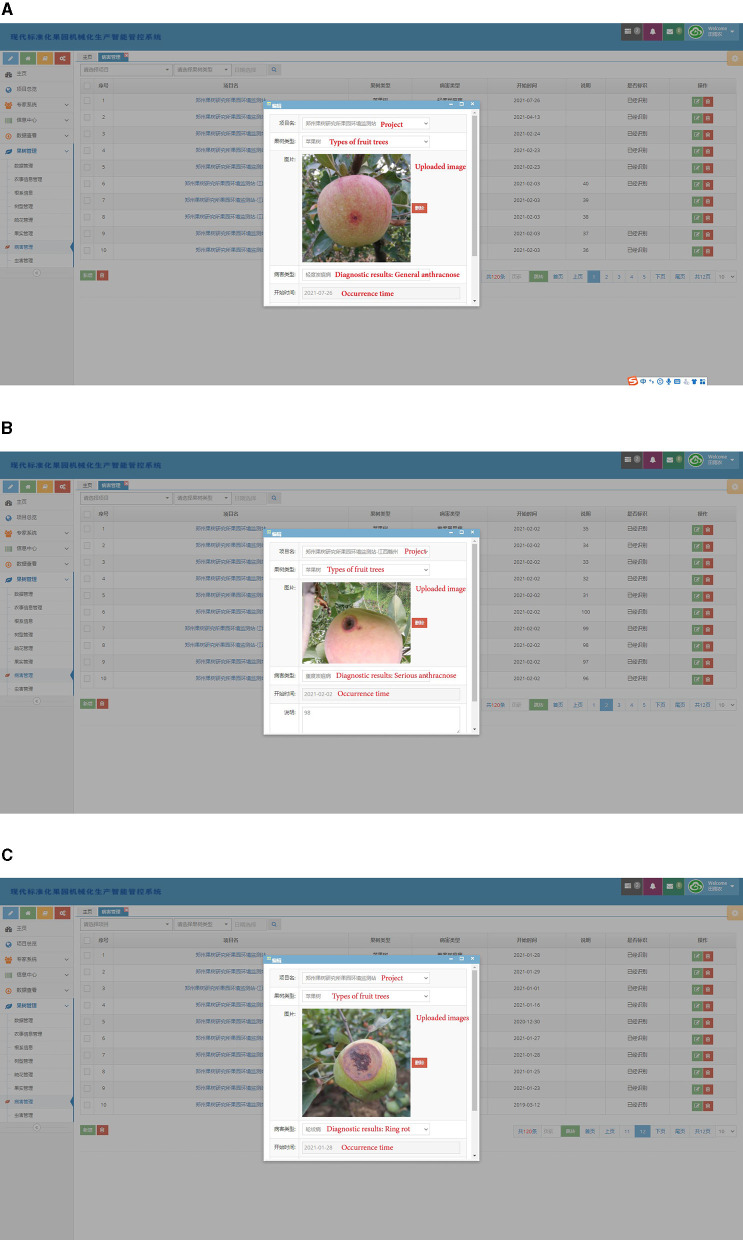
Several diagnosis results in practical application scenarios. **(A)** General anthracnose, **(B)** serious anthracnose, **(C)** ring rot.

In order to apply the disease diagnosis model in the actual scenario, a related disease management software is developed. The software consists of a human-computer interface on web page and a cloud data processing system. The Multi-scale Dense Inception-ResNet-V2 classification model is deployed in the cloud data processing system. In practice, the orchard staff can upload the disease images collected in field through the human-computer interface. The cloud system obtains the diagnosis results by processing the uploaded images and gives feedback to the human-computer interface. Some of the diagnostic results are shown in Figure 10. In practical application scenarios, 54 disease images collected in orchards were diagnosed, and the diagnostic accuracy was 94.44%. With the continuous expansion of the disease image datasets, the diagnosis model can also identify more kinds of diseases, which will further reflects the generalization ability.

## 4. Conclusions

A Multi-scale Dense network architecture is proposed in this paper to diagnose images of 11 categories including healthy apple leaves and fruits and typical diseased apple leaves and fruits. The diagnosis results have reached the state-of-the-art performance. The detailed work is summarized as follows.

1. Aiming at the problem of insufficient image data for general anthracnose, serious anthracnose, and ring rot, the Cycle-GAN method is adopted to learn the image features of healthy apples and diseased apples, and generates different categories of the lesions on the surface of healthy apple images. Experiments have verified that the generated images have greatly enriched the image dataset. The Cycle-GAN technique outperforms traditional image augmentation methods.
2. The DenseNet and Multi-scale connection method are employed to improve the existing deep learning models, and the Multi-scale Dense Inception-V4 and Multi-scale Dense Inception-ResNet-V2 classification models are proposed. Experiments have shown that the detection accuracy of the two proposed models have reached 94.31 and 94.74%, respectively, which not only improves the classification results of the original models, but also achieves the state-of-the-art diagnosis performance.

In future work, we will collect more images of different types of apple diseases and analyze the image characteristics of different diseases. The automatic diagnosis ability will be further enhanced through dataset expansion and model improvement.

## Data Availability Statement

The original contributions presented in the study are included in the article/Supplementary Material, further inquiries can be directed to the corresponding author/s.

## Author Contributions

YT was responsible for methodological research and paper writing. EL and MT guided the research ideas and framework of the paper. ZL was responsible for the research of disease data augmentation. XH provided the raw data for the experiments. All authors contributed to the article and approved the submitted version.

## Funding

This work was supported by the National Key Research and Development Plan (2017YFC0806501), National Natural Science Foundation (U1713224 and 61973300), and the Science and Technology Innovation Project of Beijing (Z181100003818007).

## Conflict of Interest

The authors declare that the research was conducted in the absence of any commercial or financial relationships that could be construed as a potential conflict of interest.

## Publisher's Note

All claims expressed in this article are solely those of the authors and do not necessarily represent those of their affiliated organizations, or those of the publisher, the editors and the reviewers. Any product that may be evaluated in this article, or claim that may be made by its manufacturer, is not guaranteed or endorsed by the publisher.
